# Organ-Confined Giant Renal Cell Carcinoma: A Case Report and Literature Review

**DOI:** 10.7759/cureus.115270

**Published:** 2026-08-26

**Authors:** Saket Singh, Jeetendra Jaidiya, Vinayaka H J, Rakesh Kumar, Bibin Sunny

**Affiliations:** 1 Urology, All India Institute of Medical Sciences, Patna, IND

**Keywords:** clear cell carcinoma, giant renal tumor, organ confined renal tumor, radical nephrectomy, renal cell carcinoma

## Abstract

Giant renal cell carcinoma (RCC) is a rare clinical entity with no universally accepted definition, typically used to describe exceptionally large renal tumors. With the widespread use of cross-sectional imaging, such massive tumors are increasingly uncommon. Tumor size is generally considered a prognostic factor; however, its correlation with biological behavior remains variable.

We report the case of a 53-year-old female who presented with progressive abdominal distension over two years, associated with recent-onset abdominal discomfort and constipation. Clinical examination revealed a large, firm, non-tender abdominal mass occupying the left hemiabdomen and crossing the midline. Contrast-enhanced computed tomography demonstrated a large heterogeneous mass arising from the left kidney, measuring 25 cm × 23 cm × 18 cm, with no evidence of vascular invasion, lymph node involvement, or distant metastasis. The patient underwent left open radical nephrectomy via a midline transperitoneal approach. Intraoperatively, a massive renal tumor was identified, with challenging hilar dissection due to its size. The renal vessels were successfully controlled, and the tumor was excised en bloc. Enlarged para-aortic lymph nodes were removed. Histopathological examination confirmed clear cell RCC, staged as pT2bN0M0. The resected tumor weighed approximately 8.2 kg. Lymph nodes were negative for metastasis. The postoperative course was uneventful, and no adjuvant therapy was administered.

This case highlights that even extremely large RCCs may remain organ-confined without nodal or distant metastasis, demonstrating a discordance between tumor size and biological aggressiveness. It underscores that tumor size alone may not reliably predict metastatic potential and supports the role of upfront radical nephrectomy as an effective treatment strategy in selected patients with giant but resectable tumors.

## Introduction

Renal cell carcinoma (RCC) is the most common malignant tumor arising from the kidney and accounts for approximately 2%-3% of all adult malignant neoplasms; it is also considered the most lethal among common urologic cancers [1,2]. Annually, around 76,000 new cases are diagnosed in the United States, with approximately 430,000 cases identified globally, resulting in about 16,000 and 130,000 deaths, respectively [1]. The global incidence is estimated at 16 new cases per 100,000 individuals per year, with a male-to-female ratio of nearly 2:1 [1]. RCC predominantly affects older adults, typically presenting between 55 and 75 years of age, with a median age at diagnosis of approximately 64 years. Since the 1970s, the incidence of RCC has increased by approximately 3% annually, largely attributed to the widespread use of ultrasonography and computed tomography (CT) for evaluating nonspecific abdominal symptoms [3]. The majority of RCC cases are sporadic, occurring without an identifiable inherited genetic predisposition, while 4%-6% are hereditary [4,5]. Tumor size is a well-established prognostic factor in RCC, serving as an independent predictor of outcomes for both organ-confined and locally advanced disease [6,7]. Although this is partly due to its strong association with pathological tumor stage, several studies have demonstrated that tumor size itself holds prognostic significance [6,8,9]. Larger tumors are more frequently associated with clear-cell histology and higher nuclear grade, both of which indicate a poorer prognosis [10,11]. Clear-cell RCC is the most common histological subtype of RCC, while nuclear grade reflects the microscopic characteristics of tumor cells and provides prognostic information regarding tumor aggressiveness. The term "giant renal cell carcinoma" is not standardized in the literature and is variably used to describe exceptionally large renal tumors, typically those exceeding 20 cm or several kilograms in weight. Such cases are rarely encountered due to the typically indolent growth of RCC and the increasing detection of smaller tumors through modern imaging. We report a rare case of a giant RCC originating from the left kidney, measuring 25 cm × 23 cm × 18 cm and weighing 8200 g (approximately 18.1 lb). This case highlights a rare presentation of giant RCC with an unusual discordance between tumor size and biological behavior, as the tumor remained organ-confined despite its massive dimensions. It further underscores that tumor size alone may not reliably predict aggressiveness or metastatic potential. Additionally, it demonstrates that upfront radical nephrectomy remains feasible and effective even in extreme tumor presentations.

## Case presentation

The patient was a 53-year-old female with a two-year history of gradually progressive abdominal distension, recently associated with abdominal discomfort and constipation. She had no history of hematuria or flank pain, the other two components of the classical triad of renal tumors. Examination revealed a firm, non-tender mass in the left abdomen crossing the midline, with restricted mobility. No abnormalities were detected on systemic examination. She had no known comorbidities or significant family history. Hematological evaluation revealed a hemoglobin level of 10.1 g/dL. Serum renal and liver function tests were within normal limits.

**Figure 1:**
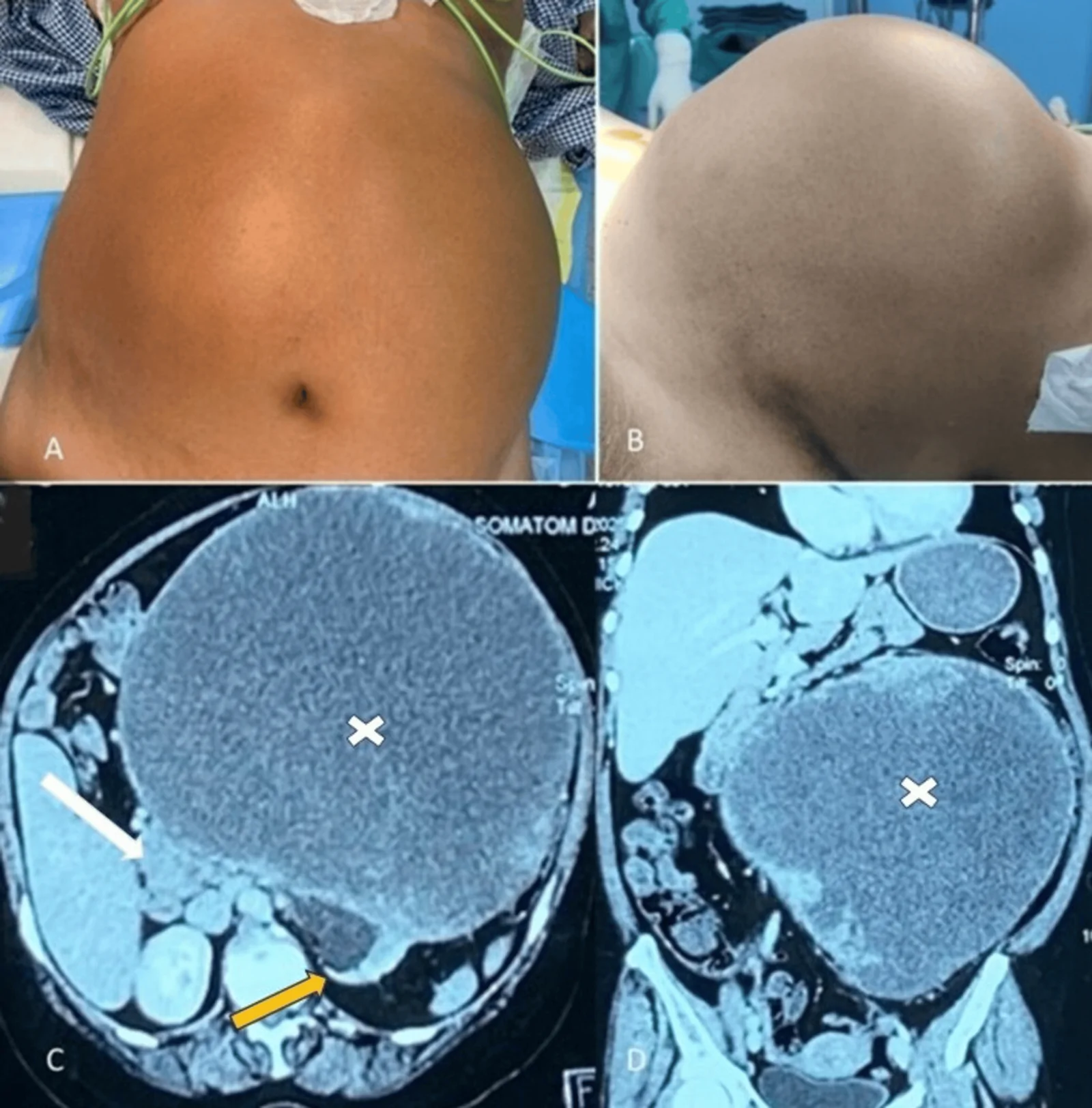
A, B) Preoperative abdominal photographs demonstrating the large abdominal mass; C) axial and D) coronal CECT images demonstrating the giant renal mass (white crosses). The yellow arrow indicates the left kidney, from which the mass originates, and the white arrow indicates displacement of the adjacent abdominal viscera CECT: contrast-enhanced computed tomography

Abdominopelvic ultrasonography revealed a large heterogeneous mass measuring 18 cm × 16 cm arising from the lower pole of the left kidney. CT urography demonstrated a large heterogeneous, predominantly non-enhancing lesion with enhancing solid components arising from the lower and mid poles of the left kidney, measuring 25 cm × 23 cm × 18 cm. There was no evidence of direct invasion of the renal vein or inferior vena cava, and no lymph node metastasis was observed. Chest CT was negative for metastatic disease (Figure 1).

The patient was counselled regarding the planned surgical procedure and associated perioperative risks, and written informed consent was obtained. She subsequently underwent open left radical nephrectomy through a midline transperitoneal approach. Upon entering the peritoneal cavity, a massive retroperitoneal tumor arising from the left kidney was identified. The descending colon was mobilized medially from the rectosigmoid junction to the splenic flexure, facilitating circumferential dissection of the tumor from the surrounding structures and overlying mesentery.

Identification of the renal hilum was technically challenging because of the extreme size and weight of the tumor. The markedly dilated left gonadal vessels were identified and traced cranially to facilitate localization of the renal hilum. A single markedly dilated renal vein and a single normal-caliber renal artery were identified. The renal vein was carefully examined up to its junction with the inferior vena cava, with no evidence of tumor thrombus. The renal artery was clipped and divided, followed by secure closure and division of the renal vein using polypropylene sutures. The ureter was subsequently clipped and divided, and the kidney with the tumor was carefully mobilized from the surrounding structures and removed en bloc.

**Figure 2:**
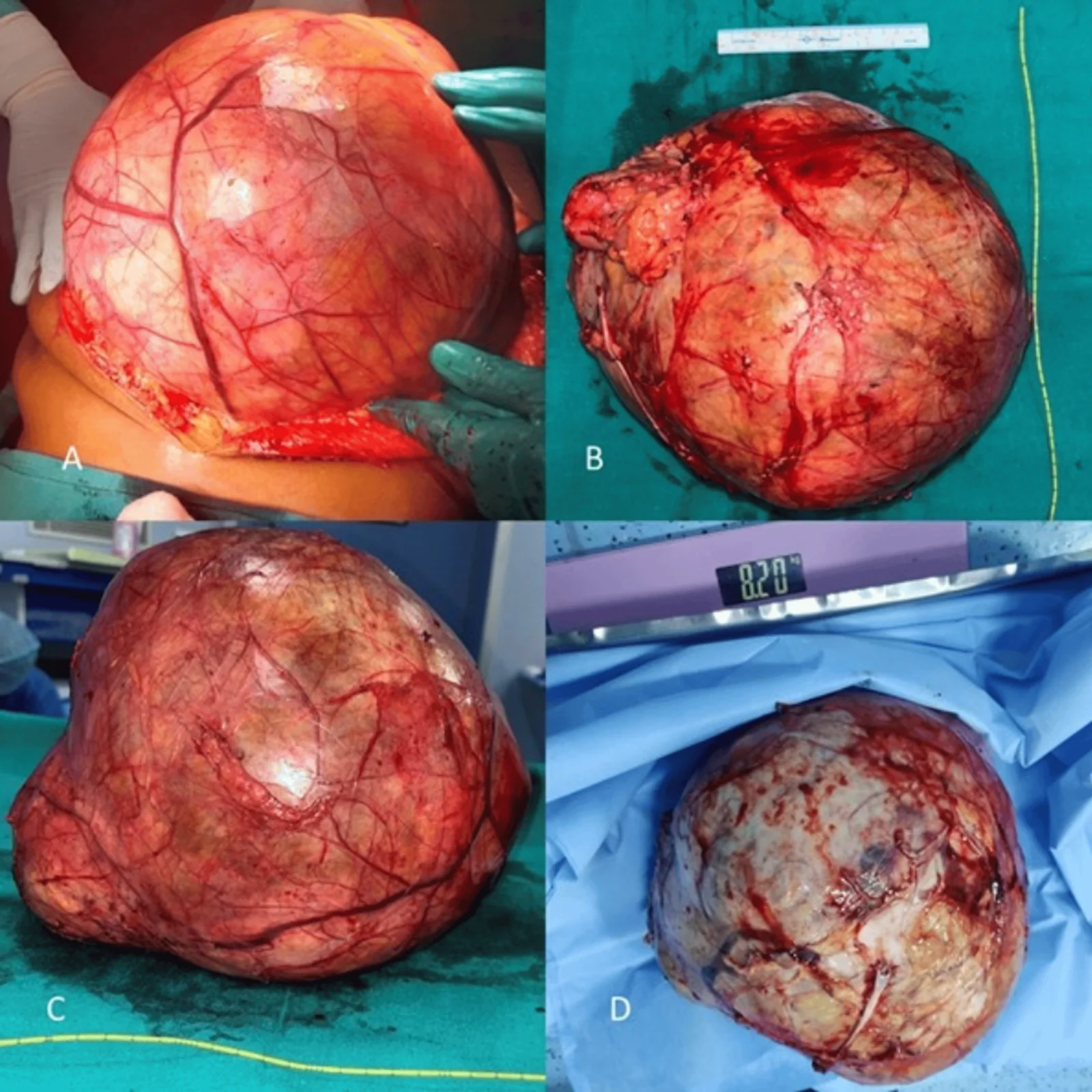
A) Intraoperative image of the left renal mass; B, C, D) Gross specimen images after left radical nephrectomy showing its various dimensions and weight

The resected specimen measured approximately 30 cm × 28 cm and weighed 8.2 kg immediately after surgery (Figure 2). The discrepancy between the in vivo radiological measurement and the ex vivo gross specimen measurement is attributable to tissue relaxation and the inclusion of surrounding perinephric tissues in the en bloc specimen. Two enlarged left para-aortic lymph nodes identified intraoperatively were excised and sent for histopathological examination. Despite compression of the left renal vein by the massive tumor, there was no evidence of renal vein thrombosis or gross invasion of adjacent structures. Meticulous hemostasis was achieved, a 24-Fr abdominal drain was placed, and the abdominal wall was closed in layers.

The postoperative period was uneventful. The urethral catheter was removed on postoperative day 2, and the abdominal drain was removed on postoperative day 3. Her postoperative hemoglobin was 9.8 g/dL, and urine output remained adequate throughout the recovery period. Histopathological examination revealed clear cell RCC, WHO/International Society of Urological Pathology (ISUP) Nuclear Grade 1, with focal tumor necrosis and no lymphovascular invasion or lymph node metastasis. According to the tumor, nodes, metastasis (TNM) classification, the tumor was staged as pT2bN0M0 (Stage II). After discussion with the patient and a medical oncologist, it was concluded that radical nephrectomy was the appropriate treatment in accordance with the National Comprehensive Cancer Network (NCCN) guidelines. No adjuvant therapy was deemed necessary at this stage. The patient was planned for NCCN guideline-based surveillance, including annual clinical and laboratory assessment, abdominal contrast-enhanced computed tomography/MRI every six months for the first two years followed by annual imaging for up to five years or longer as clinically indicated, and annual chest imaging for at least five years.

## Discussion

At present, there is no universally accepted definition of giant RCC based on tumor size or weight, and the term does not represent a formal TNM staging category. Rather, “giant RCC” is used as a descriptive term in the literature to refer to exceptionally large renal tumors. Numerous case reports (Table 1), including the current case, have documented exceptionally large malignant renal tumors. The majority of large renal masses are malignant neoplasms, typically subtypes of RCC such as clear cell, papillary, or chromophobe carcinoma. Takeda et al. reported the largest renal tumor globally, measuring 43 cm in diameter and weighing 13 kg [12]. With the increasing utilization of diagnostic imaging for the assessment of abdominal symptoms, the incidence of RCC has increased significantly over the past three decades, while the size of detected lesions has progressively decreased [13]. Several case reports have also suggested that giant renal masses occur more frequently in males than in females [12,14-17].

**Table 1:** Reported cases of large renal masses with histopathological diagnosis RCC: renal cell carcinoma

Case	Year	Age	Sex	Size (cm)	Weight (kg)	Diagnosis	Reference
1	2020	59	M	43	13	Papillary RCC	Takeda et al. [12]
2	2009	55	M	35	11.5	Chromophobe RCC	Suzuki et al. [14]
3	2024	50	M	35	9.2	Clear cell RCC	Kumar et al. [15]
4	2026	53	F	30	8.2	Clear cell RCC	Present case
5	2016	52	M	23	3.63	Leiomyosarcoma	Sawh et al. [16]

Most renal tumors grow slowly over time, and the risk of metastasis generally increases with tumor size. A median tumor size of 7.5 cm has been linked to a 40% higher risk of synchronous metastatic disease. It is estimated that for every 1 cm increase in tumor size, the chance of synchronous metastasis rises by about 22%-38% [17]. Regarding histologic subtype, tumors larger than 10 cm have relatively high metastatic rates in clear cell RCC (41.9%) and papillary RCC (28.6%), while chromophobe RCC shows a significantly lower rate of 11% [18]. It is believed that the low-grade nature of some RCCs allows for slow, expansive, and clinically inactive growth while remaining confined to the kidney. As a result, despite its large size, the tumor in our case was classified as pT2b according to the American Joint Committee on Cancer eighth edition [19]. The absence of distant metastasis in our patient, despite the tumor's large size, is not fully understood; however, several factors may explain this. First, there was no evidence of gross or microscopic invasion into the major renal vein, its segmental branches, or lymphovascular structures. Second, the tumor displayed an expansive rather than infiltrative growth pattern. Lastly, its low-grade histology likely contributed to the reduced metastatic potential, as low-grade carcinomas are generally less likely to metastasize than high-grade tumors [12].

From a management perspective, the role of neoadjuvant systemic therapy in RCC remains limited. Current NCCN and European Association of Urology (EAU) guidelines do not recommend neoadjuvant therapy for resectable, non-metastatic RCC [20]. Although targeted therapies such as tyrosine kinase inhibitors have shown potential for tumor downsizing in selected cases, their use remains investigational, with no proven survival benefit in the neoadjuvant setting. Neoadjuvant therapy is generally reserved for patients with unresectable disease, significant inferior vena cava thrombus, or metastatic RCC.

In the present case, the tumor was deemed resectable on preoperative imaging, with no evidence of distant metastasis or major vascular invasion. Therefore, upfront radical nephrectomy was performed in accordance with established guidelines. Despite the technical challenges posed by the tumor size, complete surgical excision was achieved with an uneventful postoperative recovery, underscoring that surgery remains the cornerstone of treatment even in extreme presentations.

This case adds to the limited literature on giant RCC and reinforces two key points: first, that massive tumor size does not invariably correlate with advanced or metastatic disease; and second, that appropriately selected patients can achieve favorable outcomes with timely surgical intervention without the need for neoadjuvant therapy. The absence of long-term follow-up and the inherent limitations of a single case report should be considered when interpreting these findings.

## Conclusions

For large renal tumors, a midline transperitoneal incision provides optimal exposure, facilitating the safe mobilization of the colon and access to the retroperitoneal space. Preoperative imaging is crucial for estimating tumor size, vascular anatomy, and potential adhesions, thereby aiding in determining the appropriate incision length and surgical approach. The presence of large renal masses can complicate the identification of the renal hilum. Tracing the path of dilated gonadal vessels to the hilum can assist in the secure management of the renal vessels. It is imperative to thoroughly inspect the renal vein up to the inferior vena cava to rule out tumor thrombus. Mobilization of the transverse and descending colon aids in the circumferential dissection of the tumor. Low-grade, expansile tumors may allow for careful separation from surrounding tissues without infiltration, underscoring the importance of gentle tissue handling. Ensuring meticulous hemostasis is essential.
